# Chromatin remodeling during *in vivo* neural stem cells differentiating to neurons in early *Drosophila* embryos

**DOI:** 10.1038/cdd.2016.135

**Published:** 2016-11-18

**Authors:** Youqiong Ye, Min Li, Liang Gu, Xiaolong Chen, Jiejun Shi, Xiaobai Zhang, Cizhong Jiang

**Affiliations:** 1 Department of Clinical Laboratory Medicine, Shanghai Tenth People's Hospital of Tongji University, Shanghai Key Laboratory of Signaling and Disease Research, The School of Life Sciences and Technology, The Collaborative Innovation Center for Brain Science, Tongji University, Shanghai 200092, China

## Abstract

Neurons are a key component of the nervous system and differentiate from multipotent neural stem cells (NSCs). Chromatin remodeling has a critical role in the differentiation process. However, its *in vivo* epigenetic regulatory role remains unknown. We show here that nucleosome depletion regions (NDRs) form in both proximal promoters and distal enhancers during NSCs differentiating into neurons in the early *Drosophila* embryonic development. NDR formation in the regulatory regions involves nucleosome shift and eviction. Nucleosome occupancy in promoter NDRs is inversely proportional to the gene activity. Genes with promoter NDR formation during differentiation are enriched for functions related to neuron development and maturation. Active histone-modification signals (H3K4me3 and H3K9ac) in promoters are gained in neurons in two modes: *de novo* establishment to high levels or increase from the existing levels in NSCs. The gene sets corresponding to the two modes have different neuron-related functions. Dynamic changes of H3K27ac and H3K9ac signals in enhancers and promoters synergistically repress genes associated with neural stem or progenitor cell-related pluripotency and upregulate genes associated with neuron projection morphogenesis, neuron differentiation, and so on. Our results offer new insights into chromatin remodeling during *in vivo* neuron development and lay a foundation for its epigenetic regulatory mechanism study of other lineage specification.

Neurons are a major and important cell type of the nervous system. Neurons differentiate from multipotent precursors called neural stem cells (NSCs). This indicates that an extensive epigenomic change occurs during differentiation.^1^ Epigenomic signatures, including chromatin accessibility, histone modifications (HMs), and DNA methylation, regulate the gene activity. The abnormal epigenomic changes disrupt genes that function in neural development and consequently lead to neurological and psychiatric diseases. Therefore, it is a prerequisite to characterize the *in vivo* chromatin states of neurons and NSCs for the analysis of epigenomic changes during NSCs differentiating into neurons.

There are many studies on the role of chromatin remodeling in cell fate specification using *in vitro* cell differentiation platforms. Overall, differentiated cells have a more condensed chromatin structure than stem cells.^2^ A bunch of studies showed changes in histone-modification patterns in embryonic stem cell (ESC) differentiation.^3, 4, 5^ A recent study profiled chromatin state dynamics across 16 stages of hematopoietic differentiation.^6^ Global chromatin states of mouse ESCs, neural progenitor cells, and embryonic fibroblasts were reported, as well as new bivalent chromatin marks.^7^ Both Foxa2 and H2A.Z mediate nucleosome depletion during ESCs differentiating into the endoderm.^8^ A recent study identified 85 core HM sites in mouse ESCs and 78 sites in neural progenitor cells.^9^ To date, there is no study reported focusing on chromatin dynamics during *in vivo* neuron development.

It is critical to use specific *in vivo* cell types to investigate epigenetic reprogramming during differentiation because cell cultures or *in vitro* differentiated cells lack the proper *in vivo* context. However, it is a technical challenge to isolate specific neurons and NSCs for epigenomic studies that require a large number of cells. Manual sorting^10^ is laborious and only useful for collecting a small number of cells. Laser capture microdissection^11^ is also not suitable for isolating large number of cells required by chromatin assays. Although fluorescence-activated cell sorting (FACS) can isolate large number of cells, it requires molecular markers specific to the cell type of interest that are not always available. FACS requires harsh treatment of the tissue and does not work efficiently in complex tissues such as the brain. As such, FACS is not optimal for genome-wide epigenomic profiling. Owing to an alternate approach, INTACT (isolation of nuclei tagged in specific cell types),^12^ it introduces into *Drosophila* genome a nuclear targeting fusion (*NTF*) gene consisting of 3xFLAG, BLRP (biotin ligase recognition peptide, a preferred substrate for BirA), mCherry, and RanGap (expressed in the cytoplasm and outer nuclear envelope). The *NTF* gene is expressed under the control of a certain cell-specific gene promoter. Therefore, INTACT can capture cell-type-specific nuclei suitable for gene expression, epigenomic, and proteomic profiling within a tissue through affinity purification of the NTF protein.^13, 14, 15^ It allows the study of distinct cell types at different stages of development.

Here we applied INTACT in *Drosophila* to isolate NSCs and neurons for their epigenome profiling including transcriptome, genome-wide nucleosome occupancy, and global core HM signals. Epigenomic analyses show that nucleosome depletion regions (NDRs) form in both promoters and enhancers through nucleosome shift and eviction. Nucleosome occupancy in promoter NDRs is inversely proportional to the gene activity. Genes with promoter NDR formation during differentiation are enriched for neuron development and maturation. Active H3K4me3 and H3K9ac are deposited in the promoters of genes in neurons related to neuronal functions in two modes: *de novo* establishment or increase from existing levels in NSCs. In addition, changes of H3K27ac and H3K9ac in promoters and enhancers synergistically upregulate genes with functional enrichment for neuron differentiation and downregulate genes with functional enrichment for neural progenitor cell-related pluripotency.

## Results

### Affinity purification of NSC and neuronal nuclei

**Figure 1:**
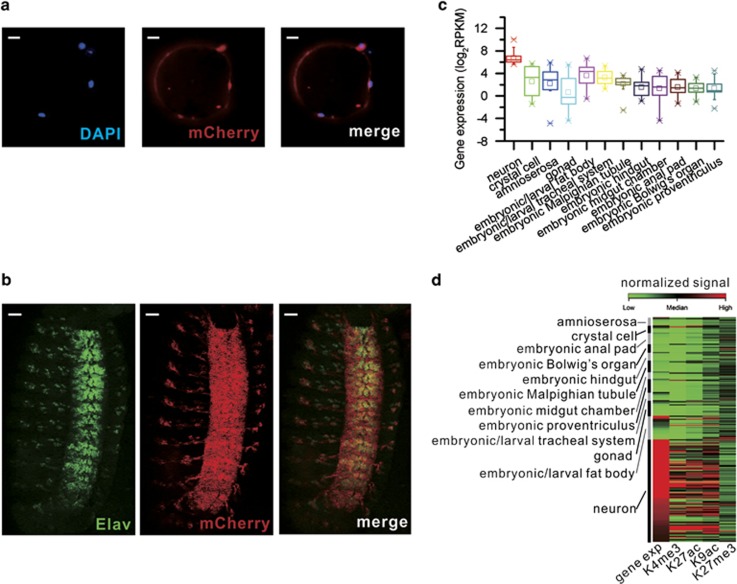
Affinity purification of neuronal nuclei from *Drosophila* *melanogaster* with the INTACT system. INTACT introduces into *Drosophila* genome an *NTF* gene consisting of 3xFLAG, BLRP (biotin ligase recognition peptide, a preferred substrate for BirA), mCherry, and RanGap (expressed in the cytoplasm and outer nuclear envelope). The *NTF* gene was expressed under the control of neuron-specific gene (*Elav*) promoter. (**a**) Three purified nuclei bound to an anti-Flag-coated bead (the large circle). Scale bar: 10 *μ*m. (**b**) Detection of mCherry (red) epitope and endogenous Elav (green) expression in a fixed stage 15 embryo (*Elav-Gal4*>*UAS-NTF*). Scale bar: 30 *μ*m. (**c**) The expression levels of neuron-specific genes are significantly higher compared with other tissue-specific genes in the purified neuronal nuclei. All *P*-values are <0.01 (Wilcoxon's rank-sum test). (**d**) Heatmap shows the profiles of core HMs in the promoter regions (±1 kb of TSS) of different tissue-specific genes in the purified neuronal nuclei. Genes are sorted descendingly by the expression level within each tissue

To apply the INTACT^15^ method to isolate neuronal nuclei, we generated a *Drosophila* strain containing *Elav-Gal4>UAS-NTF* (*Elav>NTF*). *Elav* is the commonly used neuronal marker gene.^16, 17^ The neuronal nuclei were then affinity purified from stage 15 to 16 embryos (12–14 h AEL) using anti-Flag-coated magnetic beads (Figures 1a and b). Similarly, we generated a *Drosophila* strain containing *Sca-Gal4>UAS-NTF* (*Sca>NTF*). The promoter of NSC marker gene, *Sca*,^18^ drives the specific expression of *NTF* gene in the nuclear envelope of NSCs. Therefore, NSC nuclei were collected from stage 11 embryos (5–7 h AEL) through affinity purification. Moreover, the affinity-purified neuronal and NSC nuclei reached a purity of 98±2% and 90±3%, respectively (scored >100 nuclei, average of three experiments each) (Supplementary Figure S1A).

We further examined expression profiles of different tissue-specific genes that were determined by RNA *in situ* hybridization by Berkeley *Drosophila* Genome Project.^19^ The results show that expression levels of neuronal genes are significantly higher compared with other tissue-specific genes in neuronal nuclei (Figure 1c). Similarly, expression levels of NSC-specific genes are significantly higher than other tissue-specific genes in NSC nuclei (Supplementary Figure S1B). Consistently, the active HM signals (H3K4me3, H3K27ac and H3K9ac) in promoter regions (±1 kb of transcription start sites (TSSs)) of neuronal genes are significantly higher than other tissue-specific genes in the neuronal nuclei, whereas the repressive HM signals (H3K27me3) are opposite (Figure 1d). The distribution pattern of HM signals in promoters is the same as that in NSC nuclei (Supplementary Figure S1C). These results further confirmed the purity of the isolated nuclei and appropriateness of purified nuclei for expression and chromatin profiling.

### Expression profiles of isolated NSC and neuronal nuclei

**Figure 2:**
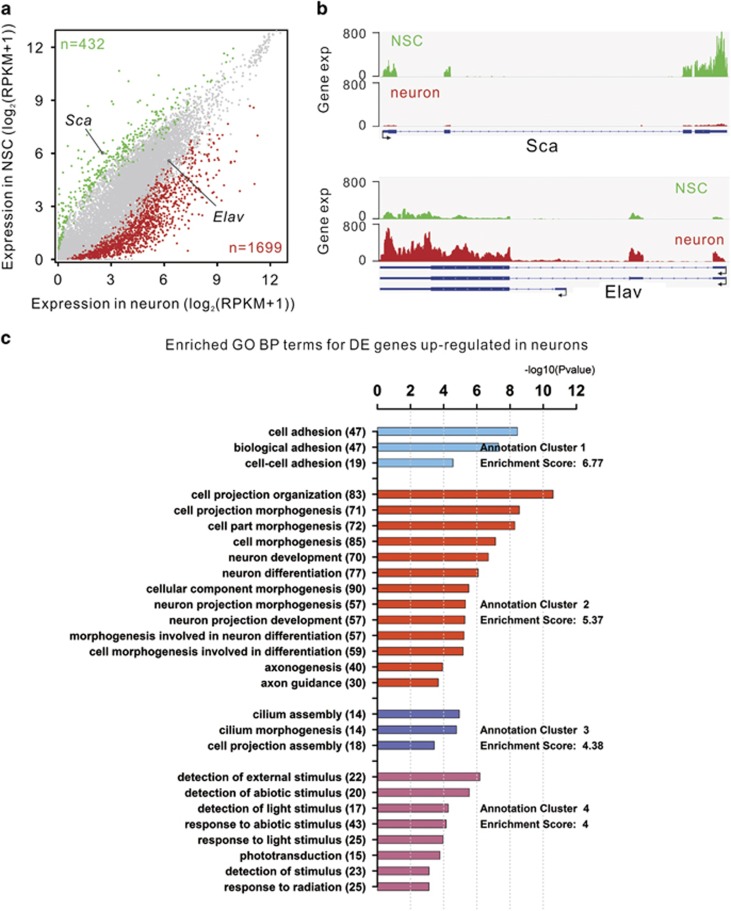
Changes of gene expression during NSCs differentiating to neurons. (**a**) Scatter plot shows the profiles of gene expression in NSCs and neurons. Significantly DEGs are indicated in colors. Green and red dots are down- and upregulated genes in neurons, respectively. The numbers of DEGs are indicated in the corners. The NSC- and neuron-marker genes (*Sca* and *Elav*) are labeled. (**b**) Read density profile for expression levels of genes *Sca* and *Elav*. (**c**) Significantly enriched GO terms for the DEGs upregulated in neurons. Each set of color bars represent a GO term cluster

We exploited RNA-seq to analyze gene expression changes during NSCs differentiating into neurons. Gene set enrichment analysis shows that genes associated with the stemness of neural progenitor cells maintain high expression levels in NSCs and are significantly downregulated in neuronal cells, whereas genes associated with neuron-related functions are significantly upregulated during differentiation (Supplementary Figure S2A). There are a total of 2131 significantly differentially expressed genes (DEGs) in neuronal cells, of which 432 are downregulated genes and 1699 are upregulated genes (Figure 2a). The NSC marker gene, *Sca*, is significantly downregulated, whereas the neuronal marker gene, *Elav*, is upregulated during the development of NSCs to neurons (Figure 2b). Gene ontology (GO) functional annotation of the DEGs found that the downregulated DEGs are mainly enriched for neural progenitor cell-related functions: cell cycle, cell division, DNA repair, cell fate commitment, neuroblast fate determination, and so on (Supplementary Figure S2B). Conversely, the upregulated DEGs have enrichment for neuron-related functions such as neuron development, neuron projection morphogenesis, axonogenesis, and so on (Figure 2c). These results indicate an extensive change in gene expression program to satisfy the different functions of these two neural cell types during differentiation.

### Global nucleosome positioning dynamics during differentiation

We next explored nucleosome positioning dynamics during differentiation. We scanned the genome with a 500-bp scan window (approximately three nucleosomes plus two linker regions) and calculated read count in each window as the nucleosome occupancy. Nucleosome occupancy changes spread out across the genome during differentiation and are statistically significant (Supplementary Figure S3A). We obtained consistent results using 150-bp (mononucleosome) and 300-bp (approximately dinucleosome plus a linker region) scan window (data not shown).

**Figure 3:**
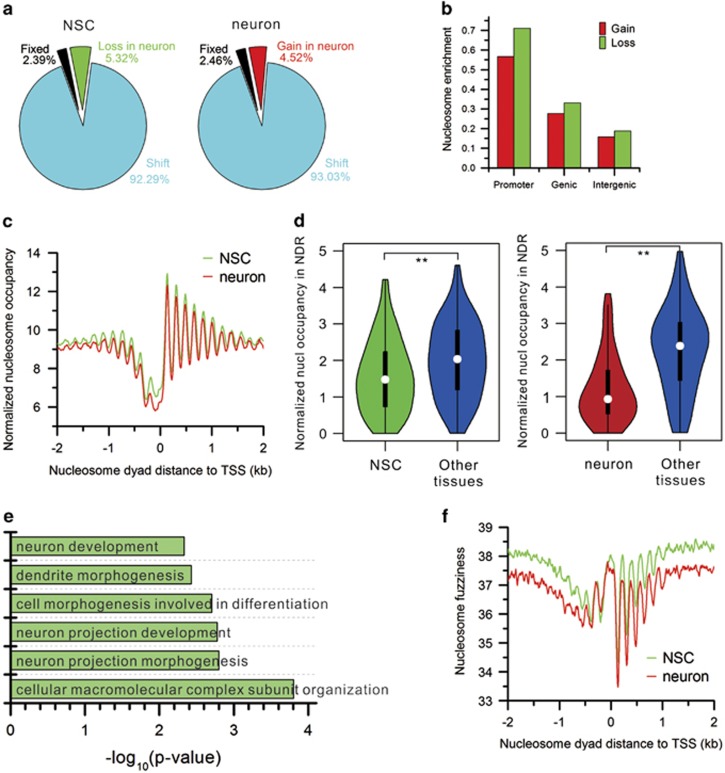
Nucleosome landscape changes. (**a**) Pie charts show the portions of fixed nucleosome, shift, loss, and gain of nucleosome. Fixed nucleosomes share the same positions in NSCs and neurons. Shift nucleosomes include the nucleosome pairs from NSCs and neurons with overlapping 20 bp or more. Nucleosome loss and gain consists of the rest of nucleosomes. (**b**) Enrichment of nucleosome gain and loss in different genomic features. The enrichment of nucleosome in promoters equals the number of nucleosomes in promoters normalized by the length of promoters. It is calculated in the same way for genic and intergenic regions. (**c**) Distribution of nucleosome locations relative to TSSs in the two cell types. Distances to TSS were binned in 10-bp intervals, then nucleosomal read count in each bin was normalized to the total number of uniquely mapped reads in millions, and finally plotted as a smoothed distribution using a moving average of three bins. (**d**) Nucleosome occupancy in the promoter NDRs (−200 and +50 bp to TSSs) in NSC-, neuron-, and other tissue-specific genes (**P<0.01, Wilcoxon's rank-sum test). Other tissues include all the non-neuron tissues in Figure 1c and non-NSC tissues in Supplementary Figure S1B, respectively. (**e**) Significantly enriched GO terms for the genes with a promoter NDR in neurons. (**f**) Distribution of nucleosome fuzziness relative to TSSs. Fuzziness measures delocalization of a nucleosome positioning, which is calculated as the standard deviation of all read coordinates that contribute to a nucleosome location

To examine nucleosome positioning dynamics, we predicted genome-wide nucleosome positions using GeneTrack,^20^ and obtained 523 516 nucleosomes in NSCs and 514 910 nucleosomes in neurons. The majority of nucleosomes (>92%) shift by 1 to 126 bp and only ~2% of nucleosomes do not shift during differentiation. In total, 5.3% of nucleosomes are lost in neurons, whereas 4.5% of nucleosomes are newly formed in neurons (Figure 3a). Interestingly, nucleosome gain and loss are enriched in promoters (Figure 3b). This implies that nucleosome remodeling in promoters may have a critical role in the transition of gene expression program during differentiation. The genes with nucleosome gain or loss in promoters are provided in Supplementary Table S2.

### Nucleosome depletion in promoter regions upon differentiation

To discover the role of nucleosome remodeling in promoter regions in transcription regulation during the differentiation, we focused on the changes in the nucleosome organization around TSS. There is a −1, NDR, +1, +2, +3, and so on canonical nucleosome arrangement around TSS (Figure 3c). We further clustered the nucleosome organization difference around TSS. The results revealed five distinct patterns for nucleosome organization change upon differentiation (Supplementary Figure S3B). Approximately 39.3% of genes maintain a similar canonical nucleosome arrangement around TSS (Cluster 2). In contrast, the periodicity of nucleosome positioning around TSS is disrupted in a set of genes (~14.8%) in neurons by a weak peak at +4 nucleosome location. Moreover, the downstream nucleosomes starting at +5 nucleosome shift toward 5′ (Cluster 5). Similarly, the downstream nucleosomes starting at +3 nucleosome shift toward 5′ but with unbroken periodicity in another set of genes (~15.8%) in neurons (Cluster 4). The rest of the genes have periodical nucleosome positioning around TSS in both cell types. However, TSS is fully (Cluster 3, ~17.0% of genes) or partially (Cluster 1, ~13.1% of genes) occupied by a nucleosome in NSCs, whereas the canonical nucleosome arrangement forms around TSS in neurons leading to exposed TSS.

Interestingly, careful examination uncovered that the nucleosome occupancy in NDRs (−200 and +50 bp to TSS) is higher in NSCs than in neurons (Figure 3c). It has been reported that nucleosome remodeling near TSS has a critical role in gene expression regulation during mouse ESCs differentiating into the lineage-committed endoderm/hepatic progenitor cells.^8^ Therefore, we analyzed the impact of nucleosome remodeling in NDRs on gene expression. The results show that nucleosome occupancy levels are negatively correlated with gene expression levels (Supplementary Figure S3C). Interestingly, nucleosome occupancy in NDRs in NSCs is significantly lower in NSC-specific genes than in other tissue-specific genes. Similarly, nucleosome occupancy in NDRs in neurons is significantly lower in neuron-specific genes than in other tissue-specific genes (Figure 3d). Consistently, gene expression levels are significantly higher in NSC-specific genes in NSCs and neuron-specific genes in neurons than in other tissue-specific genes (Figure 1c and Supplementary Figure S1B). GO term analysis of genes whose NDRs are nucleosome-depleted in NSCs but nucleosome-occupied in neurons identified enrichment for DNA replication, DNA metabolic process, and so on (Supplementary Figure S3D). In contrast, genes whose NDRs are nucleosome-depleted in neurons but nucleosome-occupied in NSCs are enriched for neuron-related GO terms such as neuron development, dendrite morphogenesis, neuron projection development, and so on (Figure 3e). This suggests that nucleosome remodeling near TSS activates corresponding lineage-commitment genes of each cell type through nucleosome depletion in NDRs during the differentiation. In addition, nucleosome fuzziness around TSS is much higher in NSCs compared with that in neurons (Figure 3f). That is, nucleosome positioning is more delocalized in NSCs than in neurons.

### Dynamics of HMs in promoter regions

HMs function in many fundamental biological processes.^21^ To understand how HMs alter and the resultant influence on gene expression during the differentiation, we first profiled HM signals in promoter regions (±1 kb of TSS). H3K4me3 levels in promoter regions take on a bimodal distribution (Supplementary Figure S4A). The smallest level value between the two peaks is selected as the threshold. The promoters with H3K4me3 level higher than the threshold are considered marked by H3K4me3. The thresholds for other HMs were determined in a similar manner (see Materials and Methods for details). Thus, HM status in each promoter is defined by combinatorial HMs. Bivalent promoters (H3K4me3+ and H3K27me3+) are prevalent in mammalian cells.^7, 22, 23, 24^ However, we only observed a very small number of bivalent promoters in both cell types (Supplementary Figure S4B). This suggests that H3K4me3 and H3K27me3 are generally exclusive in promoter regions of *Drosophila* NSCs and neuronal cells. This is consistent with the previous finding that bivalency is not a common feature of fly embryo epigenome.^25^ Nevertheless, HM states in promoters serve as a predictive for the gene activity. Genes marked by active H3K4me3 have the highest expression levels, whereas genes marked by repressive H3K27me3 have the lowest expression levels. Expression levels of genes marked by covalent HMs overall are positively correlated to the portion of active mark(s) (Supplementary Figure S4C). A recent study showed that H3K4me1 in promoters has a role in gene repression in diverse mammalian cell types.^26^ Genes with H3K4me1 and H3K27me3 covalent marks have a very low expression level in our study. GO analysis of these genes identified enrichment for gut development, leg disc development, head segmentation, and other tissue-specific commitment (Supplementary Figures S4C and D). This indicates that the covalent H3K4me1 and H3K27me3 in promoters contribute to repression of non-neural tissue genes.

**Figure 4:**
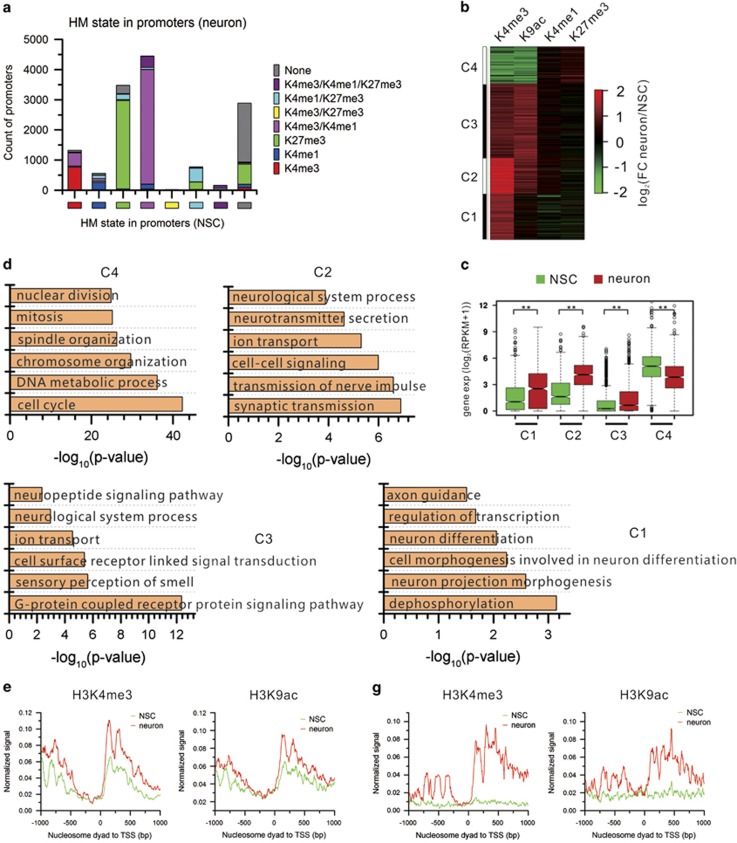

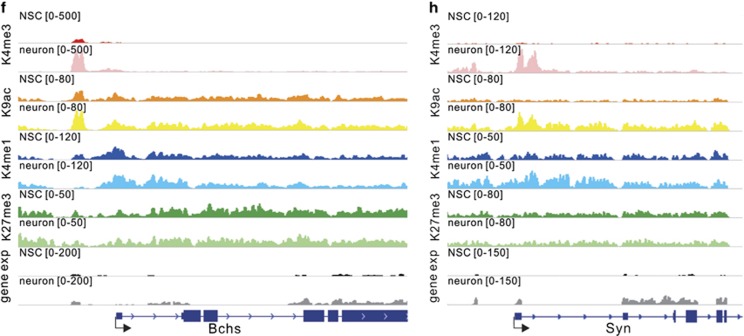
HM dynamics in promoter regions. (**a**) The count of each category of promoters defined by HM state in NSCs changing to other categories in neurons. (**b**) Clustering view of HM fold change in the promoters containing at least one HM with fold change ⩾2. (**c**). Genes in the four clusters in (**b**) are significantly differentially expressed between NSCs and neurons (**P<0.01, *T*-test). (**d**) Significantly enriched GO BP terms for the four categories of promoters in (**b**). (**e**) Increase in existing H3K4me3 and H3K9ac signals from NSCs to neurons in the promoters of gene set ‘neurological system process' (GO:0050877). (**f**) Track view of HM dynamics and the concordant expression changes of the sample gene *Bchs* for (**e**). (**g**) *De novo* gain of H3K4me3 and H3K9ac from NSCs to neurons in the promoters of gene set ‘transmission of nerve impulse' (GO:0019226). (**h**) Track view of HM dynamics and the concordant expression changes of the sample gene *Syn* for (**g**)

To understand how HMs change in promoters during the differentiation, we categorized promoters by HM content in neurons transited from promoters with a certain HM state in NSCs. The results show that the majority of promoters maintain their HM state during differentiation (Figure 4a). We further focused on the genes with one or more of HMs in their promoters that change by at least twofolds. Clustering analysis obtained four patterns of HM changes in promoters (Figure 4b). A set of genes show great decrease in active marks H3K4me3 and H3K9ac (Cluster 4). Consequently, gene expression levels are significantly downregulated in neurons (Figure 4c). These genes are enriched on the pluripotency-related GO terms such as cell cycle, DNA metabolic process, and chromatin organization (Figure 4d). On the contrary, the other two sets of genes exhibit prominent increase in H3K4me3 and H3K9ac (Clusters 3 and 2). The last set of genes show prominent increase in H3K4me3 and intermediate decrease in H3K4me1 and H3K27me3 (Cluster 1) (Figure 4b). Consistently, expression levels of these three gene sets are significantly higher in neurons than in NSCs (Figure 4c). These genes are enriched on GO terms related to neuron morphogenesis, differentiation, and function (Figure 4d).

The augmented H3K4me3 and H3K9ac signals during the differentiation could be elevated from existing marks in NSCs or *de novo* established in neurons. It is unclear if these two modes are associated with different functions. To answer this question, we examined HM changes in the promoters of different gene sets classified by GO functional annotations. Intriguingly, genes associated with functions related to neuron development gain H3K4me3 and H3K9ac in promoters upon differentiation by increase from their existing levels in NSCs (Figures 4e and f). In contrast, genes associated with neuronal functions gain H3K4me3 and H3K9ac in promoters upon differentiation through *de novo* deposition (Figures 4g and h). The repressive marks (H3K4me1 and H3K27me3) remain at an unchanged low level (Supplementary Figures S4E and F). Conversely, H3K4me3 and H3K9ac marks greatly decrease during the differentiation in genes associated with neural pluripotency, for example, notch signaling pathway (Supplementary Figures S4G and H). This suggests that different chromatin remodeling modes in promoter regions regulate the activity of genes with distinct function themes.

### Chromatin remodeling at distal regulatory elements

**Figure 5:**
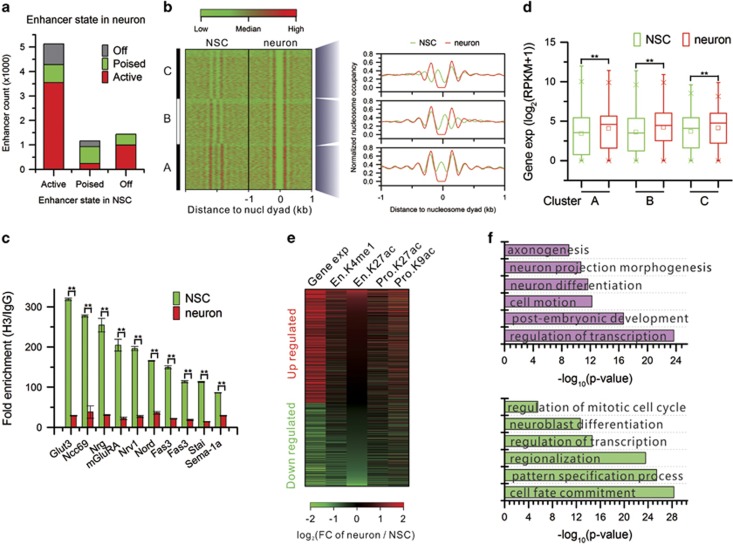
Chromatin remodeling in enhancers. (**a**) Statistical summary of each category of enhancers defined by HM state in NSCs changing to other categories in neurons. (**b**) Nucleosome organization in the regions flanking NDRs within neuron enhancers. Left heatmaps show clustering view of nucleosome occupancy in ±1 kb regions centering at NDRs in neuron enhancers. Right curve plots show the composite distribution of nucleosomes to the midpoint of the evicted nucleosomes in NDRs in neuron enhancers. (**c**) ChIP-qPCR validation for evicted nucleosomes in the enhancers of selected genes in neurons (**P<0.01, *T*-test) (**d**) Expression levels of genes associated with the three clusters of enhancers in (**b**) (**P<0.01, *T*-test). (**e**) Synergistic HM changes in enhancers and promoters. Upregulated genes have increased expression level as En.K27ac signals increase in neurons. Downregulated genes have decreased expression level as En.K27ac signals decrease in neurons. Genes are ordered in a descending order by the log 2-transformed fold change of En.K27ac. Prefixes En indicates enhancer and Pro indicates promoter. (**f**) Enriched GO terms for the upregulated (pink) and downregulated (green) genes in (**e**)

In addition to promoters, enhancers also have key roles in determining the transcriptional profile of a cell through epigenetic regulation. We first identified 7728 enhancers by H3K4me1 signals and classified enhancers by H3K27ac signals: active (H3K4me1+ and H3K27ac+), poised (H3K4me1+ and H3K27ac−), and off (H3K4me1−). Approximately 65% of enhancers are active in both cell types (Supplementary Figure S5A). As expected, expression levels of genes associated with active enhancers are significantly higher than in genes associated with poised and off enhancers (Supplementary Figure S5B). We next examined HM changes in enhancers during the differentiation. The results show that most of the active and poised enhancers in NSCs remain in the same state in neurons (Figure 5a). Seventy percent of NSC off enhancers become active in neurons. GO analysis of neuronal active enhancers transited from NSC poised and off enhancers identified enrichment for neuron differentiation, neuron projection morphogenesis, neuron recognition, and so on (Supplementary Figure S5C).

### NDR formation within neuronal enhancers through nucleosome shift and eviction

We also sought to determine whether NDRs form within enhancers during the differentiation, how they form, and their potential functions. We scanned active enhancers in neurons and collected regions that do not contain nucleosomes and are 150 bp or longer. There are total 2342 such regions that are defined as NDRs in enhancers (Figure 5b). In all, 89.5% of NDRs are 150–250 bp long indicating only one nucleosome size. Intriguingly, clustering analysis of nucleosome occupancy around NDRs by *K*-means (*K*=3) revealed three patterns of NDR formation: nucleosome eviction and nucleosome shift to 5′ or 3′ direction (Figure 5b). Chromatin immunoprecipitation-qPCR (ChIP-qPCR) results confirmed nucleosome eviction in these neuronal enhancers (Figure 5c and Supplementary Table S1). Consequently, transcription levels of genes associated with these enhancers are significantly increased from NSCs to neurons (Figure 5d). We further examined the state of these enhancers in NSCs and found that ~75% of them are active in NSCs (Supplementary Figure S5D). This implies that openness by NDR formation in these enhancers in neurons may increase DNA accessibility and have a dominant role in the activation of the target genes. Similarly, nucleosome reorganization at functional enhancers exposes conserved regulatory elements in active CD4+ T cells compared with the resting state.^27^

### Chromatin remodeling in promoters and enhancers synergistically activates NSCs differentiating to neurons

To understand how chromatin remodeling in promoters and enhancers work together to regulate the differentiation, we focused on the genes whose expression changes are positively correlated with H3K27ac signal changes during the differentiation. That is, these genes are upregulated when H3K27ac signals increase upon differentiation and *vice versa*. Remarkably, the results found that chromatin remodeling in both proximal and distal regulatory elements regulate gene expression in a synergetic manner. Namely, active HM contents increase together in both promoters and enhancers to upregulate target genes and *vice versa* (Figure 5e). GO analysis of the upregulated genes identified enrichment for axonogesis, neuron projection morphogenesis, neuron differentiation, and so on. Conversely, the downregulated genes are enriched for GO terms related to neural pluripotency or progenitor such as regulation of mitotic cell cycle, neuroblast differentiation, and so on (Figure 5f).

## Discussion

Epigenetic alterations have a critical role in cell differentiation. We profiled transcriptome, nucleosome occupancy, and core HMs in affinity-purified NSCs and neuronal cells, respectively. Epigenomics analysis found that nucleosome occupancy decrease in promoter NDRs upregulates the expression of target genes, and NDR formation in promoters facilitates NSCs differentiating into neurons. Remarkably, NDRs form in enhancers during the differentiation in two modes: nucleosome shift and eviction. As a result, it upregulates the expression of target genes and promotes differentiation. Genes gain active H3K4me3 and H3K9ac signals in promoters upon differentiation by *de novo* deposition in neurons or increase from the existing levels in NSCs. The corresponding gene sets have different neuron-related functions. Conversely, genes that lose active HMs in promoters are related to neural pluripotency and downregulated. In addition, chromatin remodeling in promoters and enhancers takes place and regulates gene expression in a synergistic manner to facilitate NSCs differentiating into neurons. Our study sheds new light on chromatin remodeling patterns and its epigenetic role in *in vivo* neuron development in early *Drosophila* embryos.

Activity control of enhancers regulates cell-type-specific patterns of gene expression and has a critical role in development.^28, 29, 30^ Nucleosome positioning is one of the key means that control the binding of transcription factors to their motifs. It has been reported that nucleosome-depleted enhancers are important to the activity of cell-type-specific regulators.^31^ Here we found that NDRs form within neuronal enhancers during differentiation. As a result, the target genes associated with these enhancers are upregulated (Figures 5b and d). This implies that NDRs within enhancers may expose *cis*-regulatory elements and facilitates binding of transcription factors. Such dynamic nucleosome positioning is composed of observations that most enhancers are cell-type-specific.^32, 33^ Notably, enhancer establishment (measured by H3K4me1 signal) is initiated earlier and can indicate the differentiation potential of progenitor cells earlier than gene expression profiles during blood formation.^6^ It will be interesting to know whether openness within enhancers (measured by NDR) can serve as an indicator of lineage commitment during *in vivo* neural development in *Drosophila*. This goal will be achieved when clean marker genes for distinct intermediate cell types during NSCs differentiating to a wide variety of mature neural cells are available.

HMs have a critical role in the development of embryonic nervous system in *Drosophila*. NSCs differentiating to glial cells require low levels of H3K9ac. High levels of CREG-binding protein (a histone acetyl-transferase) result in high levels of H3K9ac and disrupt gliogensis in *Drosophila* embryonic neural development.^34^ In contrast, our results show that H3K9ac signals in promoters are increased in neuronal cells compared with NSCs. This is consistent with the observation that neurons have relatively high levels of H3K9ac compared with most glial cells.^34^ This implies that HM contents in promoters regulate the expression of genes critical for neural cell lineage specification. Intriguingly, we further revealed that the high levels of active H3K4me3 and H3K9ac come from both *de novo* deposition in neurons and increase from existing levels in NSCs (Supplementary Figures S4E and G). Moreover, these two subclasses of genes are enriched for different neuron-related functions. The findings improve our understanding of epigenetic regulatory mechanisms underlying *Drosophila* embryonic neuronal differentiation *in vivo*.

Both promoters and enhancers are critical elements that orchestrate the regulation of gene expression during many biological processes. It has been reported that distal enhancers could be localized in close physical proximity to promoters through the formation of chromatin loops to regulate gene expression during lineage commitment and somatic cellular reprogramming.^35, 36^ Our results show that the core HMs in promoters and enhancers concordantly alter to regulate the expression of genes critical for NSCs differentiating to neurons (Figures 5d and e). However, it is unclear whether enhancer–promoter looping forms because of the involvement of other transcription factors during differentiation. Future study of genomic architecture dynamics during differentiation through circular chromatin conformation capture (4C)-based methods will fill this gap.

## Materials and Methods

### *Drosophila* strains

The transgenic line *w*^*1118*^*;p[UASRG]6* (III)^15^ was kindly provided by Professor Steven Henikoff (Fred Hutchinson Cancer Research Center, Seattle, WA, USA). This stock expresses the transgene (*NTF*) *3xFLAG-BLRP-mCherry-RanGap* and *BirA* under GAL4 driver. Other lines such as *Elav*-Gal4;Tm3/Tm6B (X;III), *Sca*-Gal4 (II) and sp/cyo;Dr./Tm6B (II;III) were obtained from the Bloomington Stock Center (Bloomington, IN, USA).

*Elav*-Gal4;Tm3/Tm6B virgin flies were crossed with *w*^*1118*^*;p[UASRG]6* male flies to generate the F1 offspring *Elav*-Gal4/+p[UASRG]6/Tm3. The F1 male flies were backcrossed with *Elav*-Gal4;Tm3/Tm6B virgin flies to generate the F2 offsprings *Elav*-Gal4;p[UASRG]6/Tm3 (female flies) and *Elav*-Gal4/+p[UASRG]6/Tm3 (male flies). Then, the F2 offsprings were self-crossed to generate the F3 offsprings that are homozygous, *Elav*-Gal4;p[UASRG]6 (female flies) and *Elav*-Gal4/+p[UASRG]6 (male flies). The embryos of F3 flies were collected by self-crossing to isolate neurons.

First, *Sca*-Gal4 virgin flies and sp/cyo;Dr/Tm6B male flies were crossed to generate the F1 offspring. We retained the ones with genotype *Sca*-Gal4/cyo;+/Tm6B (denoted as F1A) for the next cross. The sp/cyo;Dr/Tm6B virgin flies were crossed with *w*^*1118*^*;p[UASRG]6* male flies to generate the F1 offspring. We retained the ones with genotype +/cyo;p[UASRG]6/Tm6B (denoted as F1B) for the next cross. Next, F1A virgin flies were crossed with sp/cyo;Dr/Tm6B male flies to produce the F2 offspring. We retained the ones with genotype *Sca*-Gal4/cyo;Dr/Tm6B (denoted as F2A) for the next cross. The sp/cyo;Dr/Tm6B virgin flies were crossed with F1B male flies to generate the F2 offspring. We retained the ones with genotype sp/cyo;p[UASRG]6/Tm6B (denoted as F2B) for the next cross. Later, the F2A virgin flies were crossed with F2B male flies to generate the F3 offspring. We retained the ones with genotype *Sca*-Gal4/cyo;p[UASRG]6/Tm6B (denoted as F3A) for the next cross. Finally, F3A flies were self-crossed to generate the F4 offspring. We retained the ones with genotype *Sca*-Gal4/cyo;P[UASRG]6 (denoted as F4A). The embryos of F4A flies were collected by self-crossing to isolate NSCs.

### Antibodies

The following antibodies were used for ChIP: H3K4me3 (ab8580; Abcam, Cambridge, MA, USA), H3K4me1 (ab8895; Abcam), H3K9ac (ab10812; Abcam), H3K27ac (ab4729; Abcam) and H3K27me3 (ab6002; Abcam). Antibodies used for immunostaining were as follows: rat-α-Elav (from Developmental Studies Hybridoma Bank, Iowa City, IA, USA) and goat anti-rabbit IgG H&L (Alexa Fluor 488; Jackson ImmunoResearch, West Grove, PA, USA). Anti-Flag-coated M2 magnetic beads (Sigma-Aldrich, St. Louis, MO, USA) were used for pull down.

### Embryo collection and formaldehyde crosslinking

Embryos were collected on grape juice plates with a yeast paste from embryo collection cages for 2 h, and then aged at 25 °C for 3 and 10 additional hours. The harvested embryos are 5–7 and 12–14 h old, respectively. Embryos were transferred onto the mesh with PBST (PBS (137 mM NaCl, 4.3 mM Na_2_HPO_4_, 1.4 mM NaH_2_PO_4_)+0.1% Triton X-100), and were washed with tap water to remove the yeast. Then, embryos were dechorionated with 50% hypochloric acid for 3 min and were crosslinked in a 1:3 mixture of ChIP–Fixed buffer (50 mM (pH 7.6) HEPES, 100 mM NaCl, 0.1 mM EDTA, 0.5 mM EGTA) with 1.8% formaldehyde and heptane for 15 min on a shaker with a speed of 300 r.p.m. The aqueous and organic phase was replaced with PBST containing 0.25 mM glycine to cease the crosslinking reaction. The fixed embryos were rinsed three times with PBST and then stored at −80 °C for future use.

### Purification of tagged nuclei from *Drosophila* embryos

Purification of tagged nuclei was performed using the INTACT technology as described previously.^15, 37^ In brief, 0.3–0.5 g of fixed embryos were suspended in 4 ml of cold HB125 buffer (15 mM NaCl, 40 mM KCl, 15 mM (pH 7.5) Tris-HCl, 0.125 M sucrose, 0.5 mM spermidine, 0.15 mM spermine, EDTA, 0.5 mM EGTA, 1 × Complete protease inhibitor (PI)) and dounce homogenized. To monitor bead binding 0.5 ml of 5 mg/ml DAPI solution was added. The nuclei mixture was filtered through one layer of Miracloth into 50 ml conical tube and diluted to 40 ml with cold HB125 buffer; afterwards, 3 ml of OptiPrep (Sigma-Aldrich) was added and then centrifuged at 1000 × *g* for 10 min at 4 °C. The supernatant and OptiPrep cushion were discarded, leaving ~2 ml of HB125 containing nuclei concentrated at the interface. Isolated nuclei were suspended in HB125 with 60 *μ*l of anti-Flag M2 magnetic beads slurry and incubated on a rotator for 2 h at 4 °C. Beads with affinity-bound nuclei were absorbed by the magnet and then washed three times using HB125. Purified nuclei were stored at −80 °C before proceeding with ChIP.

### Immunofluorescence microscopy

Embryos were dechorionated in 50% solution of bleach and fixed in a 1:1 mixture of 4% formaldehyde in PBS with 0.3% Tween-20 and heptane for 20 min on a shaker. The aqueous phase was discarded and replaced with methanol, and embryos were shaken for 3–5 min at 300 r.p.m. to burst vitelline membranes. Embryos were rinsed three times with methanol and then rinsed three times with PBS containing 0.3% Triton X-100. Fixed embryos were blocked with PBST supplemented by 5% normal donkey serum (NDS) for 30 min and then incubated with primary antibodies of various dilutions in PBST containing 5% NDS overnight at 4 °C. These embryos were washed as described above, followed by a 1–2 h secondary antibody incubation, and then were washed three times again to avoid nonspecific binding. These stained embryos were mounted on slides with additional 50% glycerol in PBS. Slides were examined on a Zeiss Imager M2 microscopy (Goettingen, Germany). To examine bead binding, DAPI-stained total and affinity-purified nuclei were counted on a hemacytometer, respectively.

### MNase-seq and ChIP-seq

Affinity-purified nuclei were pelleted by centrifugation at 1000 × *g* for 5 min and suspended with 500 *μ*l of 37 °C preheated MNase (micrococcal nuclease) digestion buffer (10 mM (pH 7.5) Tris-HCl, 15 mM NaCl, 60 mM KCl, 2 mM CaCl_2_, 0.15 mM spermine, 0.5 mM spermidine, 1 × PI) with 12 U MNase (Worthington Biochemical Corporation, Lakewood, NJ, USA) and incubated at 37 °C for 20 min. The reaction was terminated on ice by adding EDTA to a final concentration of 10 mM for 10 min. The supernatant was discarded and the pellet was washed with A2 buffer (140 mM NaCl, 15 mM (pH 7.6) HEPES, 1 mM EDTA, 0.5 mM EGTA, 0.1% Triton X-100, 0.1% sodium deoxycholate, 1 × PI) and resuspended in A2 buffer with 0.1% SDS. The nucleosome pellet was dissolved through sonication with three cycles of 20 s duration with at least 40 s pauses between cycles at the power setting of 6 (out of 20) on a Misonix sonicator XL-2000 (Newtown, CT, USA). The supernatants with chromatin were kept for the next ChIP assay, or were reverse crosslinked to harvest nucleosomal DNA fragments as follows: chromatin was treated with RNase A at 37 °C for 0.5–1 h and followed by proteinase K treatment at 65 °C for 2 h. Later, the nucleosomal DNA was retracted by phenol–chloroform and precipitated with a 1:10:100:200 mixture of 20 mg/ml glycogen, 3 M (pH 5.3) NaOAc, nucleosomal DNA mixture, and cold 100% ethanol.

Chromatin of 10–15 *μ*g was used for each ChIP reaction with HM antibodies of various doses as described in the specifications. Mixture containing chromatin, antibody, and ChIP buffer (16.7 mM (pH 8.1) Tris-HCl, 167 mM NaCl, 1.2 mM EDTA, 1% Triton X-100, 0.01% SDS) was incubated overnight on a rotator at 4 °C. Then, 20 *μ*l of ChIP-Grade Protein G Magnetic Beads (Cell Signaling; no. 9006) was added to each IP reaction. The mixture was incubated for 2 h with rotation. Afterwards, beads were washed three times with low salt wash buffer (2 mM EDTA, 20 mM (pH 8.1) Tris-HCl, 0.1% SDS, 1% Triton X-100, 150 mM NaCl) and once with high salt wash buffer (2 mM EDTA, 20 mM (pH 8.1) Tris-HCl, 0.1% SDS, 1% Triton X-100, 500 mM NaCl) for 5 min each wash. Beads were suspended in 150 *μ*l of ChIP elution buffer (50 mM (pH 8.1) Tris-HCl, 10 mM EDTA, 0.1% SDS) at 65 °C for 45 min.

The purified mononucleosomal DNA was subjected to massively parallel DNA sequencing on Illumina HiSeq2000 platform (San Diego, CA, USA) using a 49 bp single-end protocol.

### Nuclear RNA-seq analysis

Nuclear RNA was isolated form tagged nuclei of the affinity-purified embryos without crosslinking using the RNeasy Micro Kit (Qiagen, Valencia, CA, USA). Genomic DNAs were removed with Turbo DNA-Free Kit (Ambion, Waltham, MA, USA). The RNA sequencing libraries were constructed using standard Illumina libraries prep protocols. RNA-seq was performed on Illumina HiSeq2000 platform. Sequencing reads were aligned to the *Drosophila* transcripts (FlyBase r5.43) using TopHat (v.1.3.1) with default parameter setting.^38^ The uniquely mapped reads were assembled into transcripts guided by reference annotation with Cuffdiff (v.1.3.0)^38^ to calculate gene expression levels that were normalized as fragment per kilobase per million mapped fragments. The DEGs were identified with FDR <0.05.

### Tissue-specific gene lists

Tissue-specific gene lists were downloaded from the Berkeley Drosophila Genome Project *in situ* database BDGP.^19^ Genes expressed at stages 11–12 and 13–16 in different tissue-specific cells were collected.

### Prediction of nucleosome positioning and analysis of dynamic positioning

Nucleosomal sequencing reads were aligned to the *Drosophila* reference genome (dm3) using Bowtie,^39^ allowing maximal two mismatches. The uniquely mapped reads were used to identify genome-wide nucleosome positions through the peak-calling tool GeneTrack^20^ that also calculated read count for each nucleosome. The read count was normalized by total uniquely mapped reads as the nucleosome occupancy. Nucleosome fuzziness was calculated as the standard deviation of the coordinates of all reads defining the same nucleosome as described previously.^40^ It measures how delocalized a nucleosome position is. Each nucleosome was assigned to either of promoter, genic, or intergenic regions depending on in which region the midpoint of the nucleosome was located.

Nucleosome organization change around the TSS was analyzed as in the previous study.^40^ Briefly, *Drosophila* transcript annotation was downloaded from FlyBase release 5.43. Nucleosomes located within ±1 kb of TSSs were collected. Nucleosome length equals to the fuzziness value and centers at the nucleosome midpoint that defines the nucleosome position. The region that a nucleosome length spreads out has the nucleosome occupancy. The difference in nucleosome organization around TSSs was measured by subtracting nucleosome occupancy in NSCs from that in neurons for each site. The difference clustered by *K*-means (*K*=5) and plotted as heatmap.

The original composite distribution of nucleosome around TSS of the five clusters of genes in NSCs and neurons, respectively, was calculated by aggregating nucleosomal read count at each distance relative to the TSS as follows: each read represents a nucleosome by extending toward 3′ end to a length of 147 bp. The midpoint of extended read defines the nucleosome position. We summed total read counts at each site within ±1 kb of TSSs for the five gene clusters, respectively. The nucleosome occupancy equals to the read count normalized as RPKM. We further binned the nucleosome occupancy by a 5-bp interval of nucleosome distance to TSS, and smoothed it with 5-bin moving average and 1-bin step size.

### Analysis of HM changes in the promoters

Sequencing reads were mapped to the reference genome similar to the above nucleosomal read mapping. We defined promoters as the region ±1 kb of TSSs. HM levels in a promoter were calculated as all reads within the promoter and normalized as RPKM. The density distribution of H3K4me1, H3K4me3 and H3K9ac levels in promoters is a bimodal pattern. The levels at the valley were chosen as thresholds, which was 11.6 for H3K4me1, 16 for H3K4me3, and 16 for H3K9ac. For qualitative analysis, promoters with a higher level of a certain HM compared with the threshold are characterized as marked by this HM, otherwise not. The density distribution of H3K27me3 levels in promoters is a normal distribution. We grouped promoters into two classes with or without H3K27me3 by *K*-means clustering (*K*=2).

The composite distribution of HM around TSS was calculated in the same manner as nucleosome distribution described above.

### Chromatin state in enhancers

Enhancers and their chromatin states were determined as described previously.^6^ In brief, H3K4me1-enriched regions (peaks) were identified using HOMER^41^ using a 1000-bp sliding window with a false discovery rate of 0.1%. In addition, two adjacent peaks need to be separated by at least 1000 bp to avoid redundant detection. We next combined such valid peaks from two cell types as one set of peaks by merging overlapped peaks. The peak with highest HOMER score replaces the overlapped peaks. Then, we retained total 14 234 H3K4me1 peaks for the two cell types. To remove false enhancers, we calculated H3K4me3 signals in H3K4me1 peaks and observed a bimodal distribution. A Two Gaussian mixture model was used to fit the distribution to select a threshold (=4.55). The H3K4me1 peaks with H3K4me3 levels higher than the threshold were discarded. It resulted in 7728 H3K4me1 peaks as the final set of enhancers.

To define chromatin states for enhancers, we first recalculated H3K4me1 and H3K27ac signals in the enhancers for the two cell types. We also randomly selected the same number (7728) of 1000-bp genomic regions and calculated H3K4me1 and H3K27ac signals. The smallest level of the top 10% signals was used as the false-positive threshold, which was 5.5 for H3K4me1 and 4.5 for H3K27ac, respectively. Enhancers are ‘on' with H3K4me1 level higher compared with 5.5 and ‘off' otherwise. Of ‘on' enhancers, those are ‘active' with H3K27ac level higher compared with 4.5 and ‘poised' otherwise. Finally, each enhancer was associated with a single gene based on the nearest RefSeq TSS.

### Data accession numbers

The RNA-seq, MNase-seq, and ChIP-seq data sets have been deposited in the Gene Expression Omnibus under the accession number GSE80458.

## Abbreviations

NSC: neural stem cell
NDR: nucleosome depletion region
HM: histone modification
ESC: embryonic stem cell
FACS: fluorescence-activated cell sorting
INTACT: isolation of nuclei tagged in specific cell types
NTF: nuclear targeting fusion
AEL: after egg laying
TSS: transcription start site
DEG: differentially expressed gene
GSEA: gene set enrichment analysis
GO: gene ontology
