# Supplementary material for: Chromatin remodeling during in vivo neural stem cells differentiating to neurons in early Drosophila embryos
Source: Cell Death Differ. 2016 Nov 18;24(3):409–20. doi: 10.1038/cdd.2016.135 (PMC5344203; doi:10.1038/cdd.2016.135)
Supplement: Supplementary Information [file cdd2016135x1.pdf]

Chromatin remodeling during *in vivo* neural stem cells differentiating to neurons in early *Drosophila* embryos

Youqiong Ye*, Min Li*, Liang Gu, Xiaolong Chen, Jiejun Shi, Xiaobai Zhang[#] and Cizhong Jiang[#]

Supplementary Information

Supplementary Table S1. Primers for ChIP-qPCR validation of nucleosome occupancy in NDRs in neuronal enhancers.

Associated gene	Distance to TSS (bp)	Forward primer	Reverse primer
Glut3	-6436	5'-AGCCAGCCATCTTCTCCT-3'	5'-TTTCTCGTTTCGCTCTTCTT-3'
Ncc69	-1238	5'-GGCAAGCGCCATTTGAAA-3'	5'-TGTGCGTTGCTGTATGAC-3'
Nrg	-31269	5'-TGTGTCCTCCAGTGCTAC-3'	5'-ATCTTCAGTGCTATCTACTCTG-3'
mGluRA	-2076	5'-CCTCAATCAGTCGCTTCA-3'	5'-CCCGCCAATAACTCCAAA-3'
Nrv1	-1157	5'-GCTCTGTGCTTCGGAAAT-3'	5'-AGATGCTGATGACGACGA-3'
Nord	-3748	5'-GAACCCGATCCACTAACTT-3'	5'-CAACGCCAACAACAATCA-3'
Fas3	-40803	5'-ATCGCACAGATGAGCATT-3'	5'-AGCATAAGTCCCGACAATA-3'
Fas3	-20439	5'-CTGGATCTCTGTCTGAATGG-3'	5'-CGTATAGTATCTGGCGGATG-3'
Stai	-5213	5'-TGCACATACAAGACTGACTT-3'	5'-ATATCCTGCCGTGTTCCA-3'
Sema-1a	-83351	5'-GGTTCCAGGAGCCAAGAT-3'	5'-AATGATGCCACTGAGTCTC-3'

Supplementary Table S2. Genes with nucleosome gain or loss in promoter regions during differentiation.

Genes with nucleosome gain in neurons	Genes with nucleosome loss in neurons
ato, ac, sc, mia, ial, bnk, BubR1, glu, Mcm10, polo, Sas-4, sens, feo, borr, ind, CAP-D2, dgt4, Mcm6, CG12018, cid, Cenp-C, ran, tum, gnu, CycE, Kmn1, pav, cdc2c, DNApol-alpha60, RfC4, neb, tefu, SAK, vfl, Mtor, pr-set7, Trf4-2, Ptp61F, Rca1, PPP4R2r, CycH, Bre1, SmB, Cam, Cdk8, stau, S, neur, e(r), vls, ssp3, U2af38, Pp4-19C, Hel25E, Prosbeta5, CG4203, mre11, Sry-alpha, Dim1, Apc7, mus81, fs(1)Ya, Pomp	KaiRIA, vri, klg, CG9194, tim, Rya-r44F, Ca-beta, nord, cac, cher, fusl, Fas3, CG16778, Ca-alpha1D, nwk, Syt1, rut, brp, nemy, Obp83ef, hiw, Con, Shaw, cpx, CanB, Sh, cngl, Oatp26F, slo, Oatp33Eb, tomosyn, Nmdar2, sif, ine, Timp, bchs, fray, shakB, CG11739, n-syb, Syt7, unc-104, CG42260, CG33310, Oatp74D, Gycalpha99B, t, Cirl, CG8713, CG34396, pHCl, Zip3, CG9935, Frq2, gfA, nrv3, Got2, CG10440, tutl, rdgA, Frq1, CG11655, dnc, Gld2, Tpi, moody, Sap47, CG9657, Pgk, scb, Syx4, CG12279, pyx, Fas2, nAcRalpha-34E, dare, para, Plc21C, CG5687, Nhe1, shep, acj6, ttk, Ext2, spin, CG17922, Sema-1a, Nrg, yuri, mrt, Ir, synj, Vha68-2, scramb1, synaptogyrin, wun, Tsf2, Eps-15, ey, Ca-P60A, CdsA, stmA, beta4GalNAcTA

Note: gain or loss of more than one nucleosomes may occur in a gene promoter. Here only includes genes with expression change of ≥2 folds, and associated with neural stem or progenitor cell-related pluripotency functions (left cell) such as DNA replication, cell cycle, neuroblast fate determination etc., and with neuron differentiation and maturation-related functions (right cell) such as neuron projection morphogenesis, neuron development, transmission of nerve impulse etc.

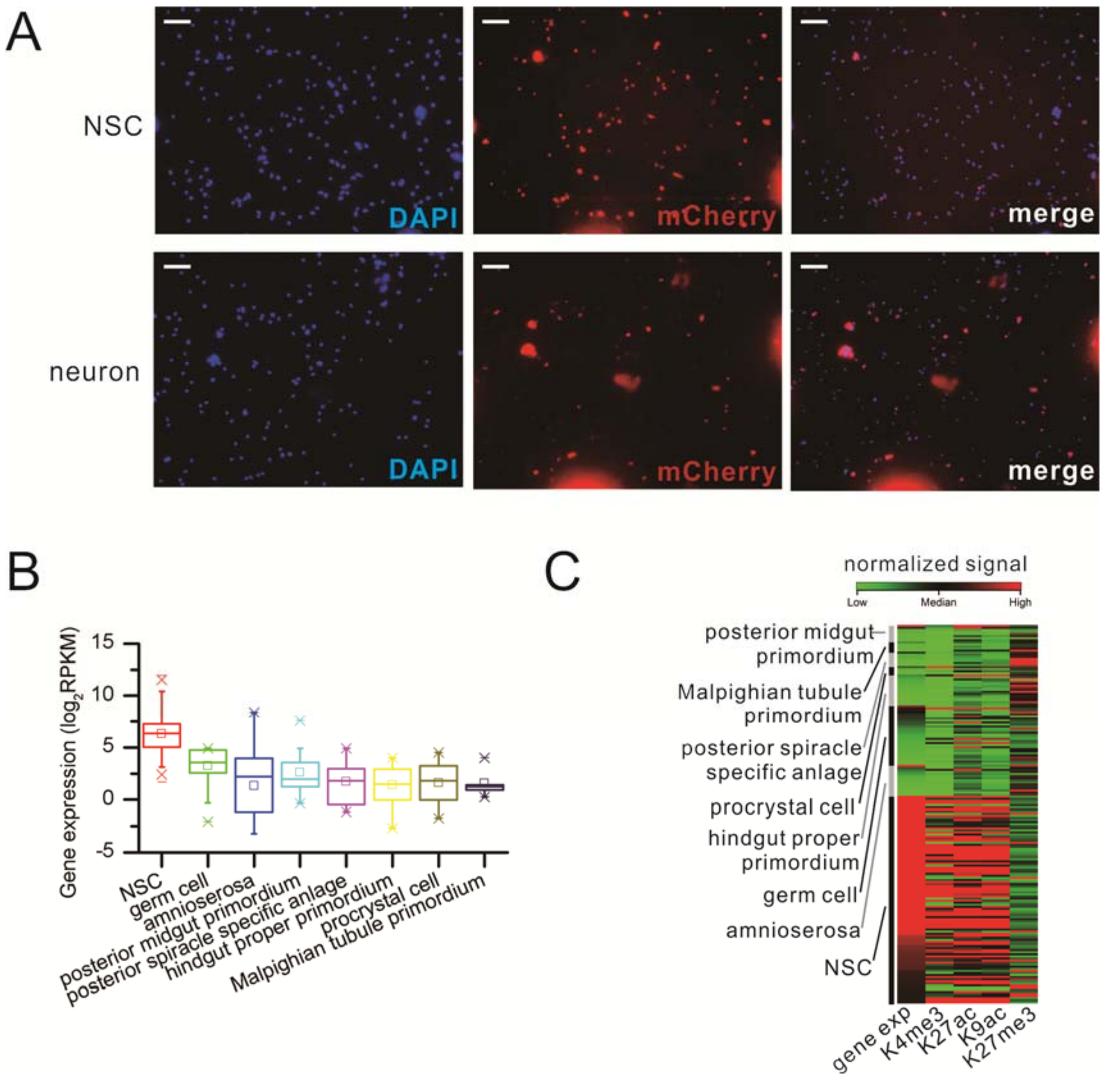

Supplementary Figure S1. Affinity purification of NSC nuclei from *D. melanogaster* with INTACT system. INTACT introduces into *Drosophila* genome a nuclear targeting fusion (NTF) gene consisting of 3xFLAG, BLRP (biotin ligase recognition peptide, a preferred substrate for BirA), mCherry, and RanGap (expressed in the cytoplasm and outer nuclear envelope). The NTF gene was expressed under the control of a cell type-specific gene promoter. **(A)** High purity of affinity captured NSC and neuronal nuclei. Scale bar: 40 µm. **(B)** The expression level of NSC-specific genes is significantly higher than other tissue-specific genes in the purified NSC nuclei. All p-values are less than 0.01 (Wilcoxon rank sum test). **(C)** Heatmap shows the profiles of core histone modifications in the promoter regions (±1 kb of TSS) of different tissue-specific genes in the purified NSC nuclei. Genes are sorted descendingly by expression level within each tissue.

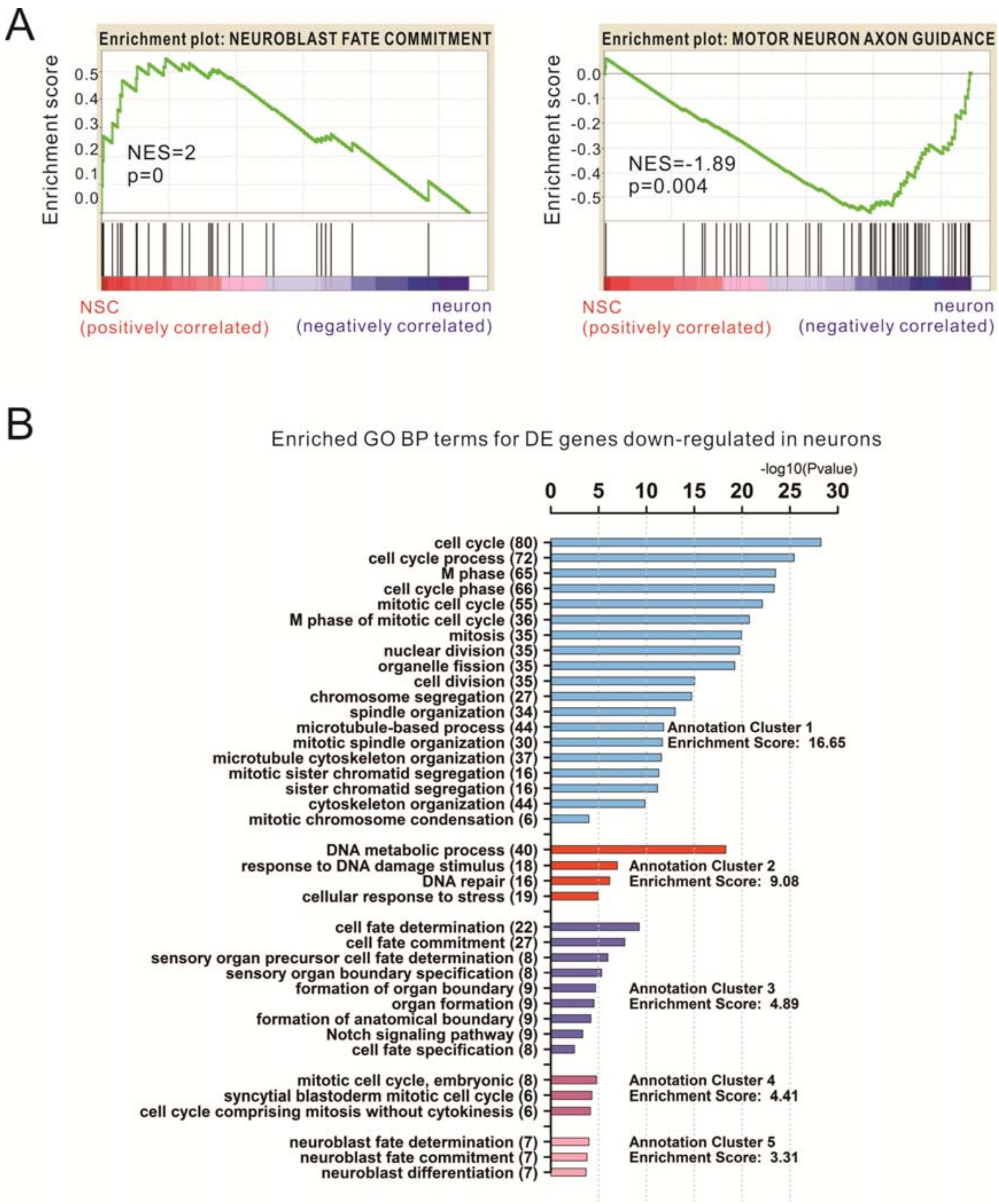

Supplementary Figure S2. Functional annotation of gene expression changes during NSCs differentiating to neurons. (A) Gene set enrichment analysis (GSEA) shows that genes with functions of "neuroblast fate commitment" are significantly down regulated in neurons, whereas genes with functions of "motor neuron axon guidance" are significantly up regulated. Normalized enrichment scores (NESs) and p-values are shown. **(B)** Significantly enriched GO terms for the DE genes down-regulated in neurons. Each set of color bars represents a GO term cluster.

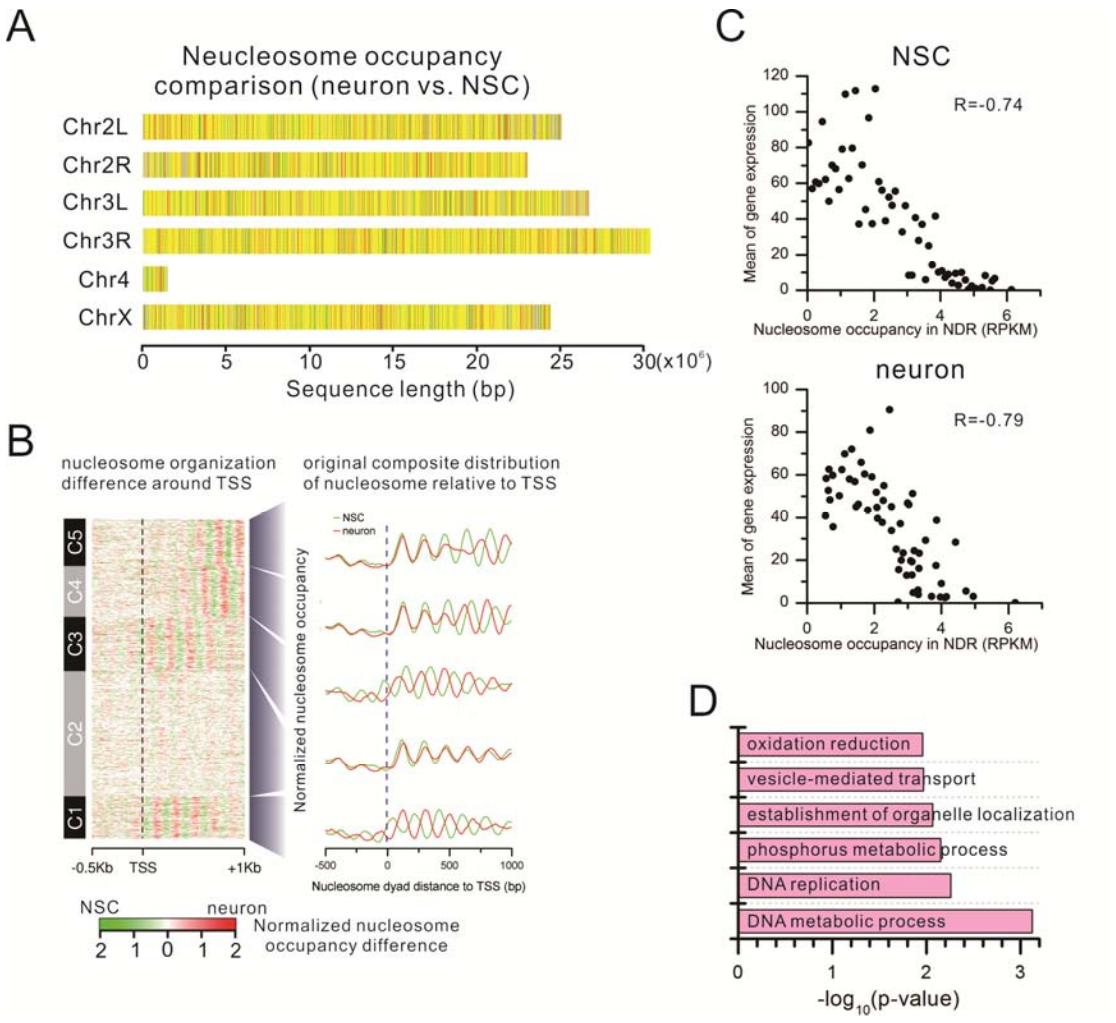

Supplementary Figure S3. Dynamic nucleosome positioning. (A) Nucleosome occupancy change across the genome by chromosomes. The genome is scanned with a 500-bp window. The ratio of nucleosome occupancy in each window is shown in colors. Red regions indicate that nucleosome occupancy in neurons is at least two-fold higher than NSCs. Green regions indicate that nucleosome occupancy in neurons is at least two-fold lower than NSCs. Yellow regions indicate that the ratio of nucleosome occupancy is less than two fold between two cell types. Grey indicates regions of Ns in the genome. The nucleosome occupancy changes are statistically significant (p-value = 6.47e-53, Student's T-test). **(B)** Altered nucleosome organization around the 5' end of genes. Left heapmap shows the changes in nucleosome arrangement around TSSs. Red and green indicates nucleosome occupancy increase and decrease in neurons compared to NSCs, respectively. Right curve plots show the original composite distribution of nucleosome locations relative to TSSs. On the basis of nucleosome occupancy change, there are five distinct patterns of altered nucleosomal phasing. The vertical dotted line indicates TSS. **(C)** Negative correlation between nucleosome occupancy in promoter NDRs and gene expression level. Pearson's correlation coefficients are given. **(D)** Significantly enriched GO terms for the genes with nucleosomes occupying in the promoter NDR in neurons.

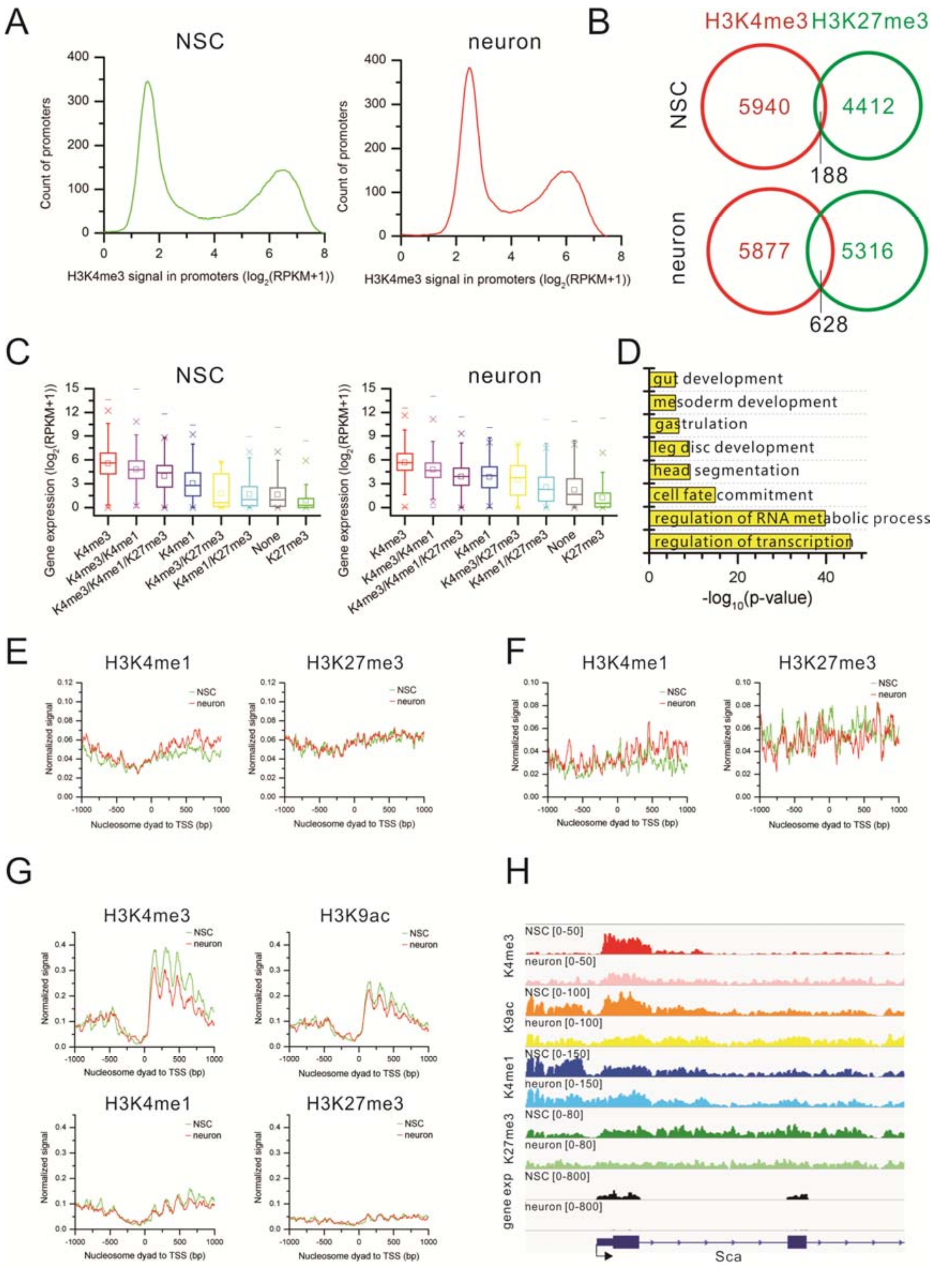

Supplementary Figure S4. Profiling analysis of histone modifications in promoter regions. **(A)** Frequency distribution of promoters based on H3K4me3 signals. **(B)** Very few H3K4me3/H3K27me3 bivalent promoters in both two cell types. **(C)** Gene expression profiles of different groups of genes classified by histone modification states. **(D)** Functional annotation analysis of genes with H3K4me1/H3K27me3 marks in their promoters in NSCs. **(E-F)** Unchanged H3K4me1 and H3K27me3 levels between the two cell types in the

promoters of gene sets “transmission of nerve impulse” (GO: GO:0019226) and “neurological system process” (GO: GO:0050877). **(G)** Profiles of core histone modifications in promoters of gene set “Notch signal pathway” (GO: GO:0007219). **(H)** Track view of HM dynamics and the concordant expression changes of the sample gene *Sca* for (G).

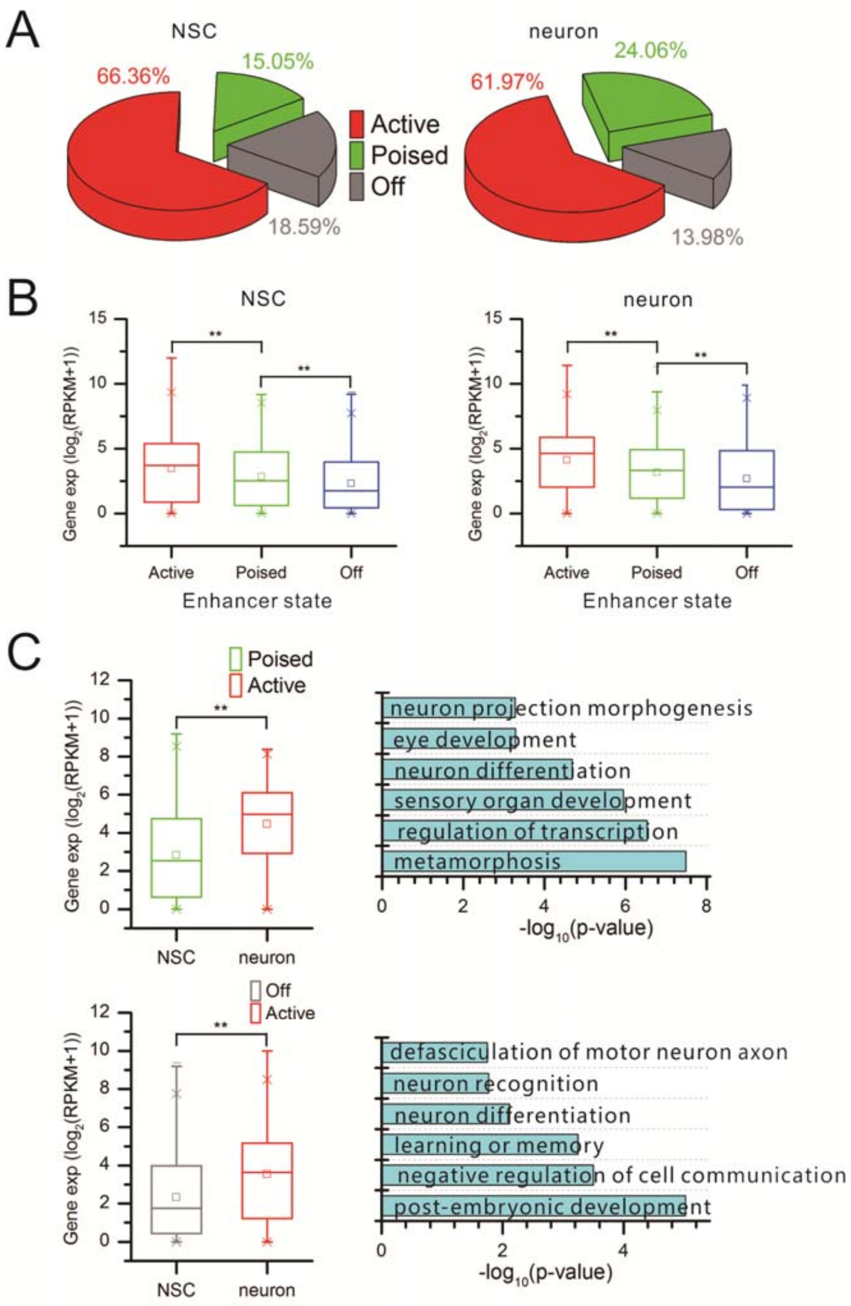

D

Count (%) of enhancers classified by different states in NSCs for each cluster of enhancers with NDR formation in neurons (enhancers in Fig. 5B)

Cluster	Active	Poised	Off
A	523 (73.6)	26 (3.7)	162 (22.8)
B	534 (78.2)	36 (5.3)	113 (16.5)
C	552 (74.0)	40 (5.4)	154 (20.6)

Supplementary Figure S5. Impact of chromatin remodeling in enhancers on gene expression. (A) Percentages of enhancers in different states. Active (H3K4me1+, H3K27ac+),

poised (H3K4me1+) and off (H3K4me1-). **(B)** Expression levels of genes associated with the three types of enhancers (**: $p < 0.01$, Wilcoxon rank sum test). **(C)** Resolution of enhancers in poised and off states in NSCs to active state in neurons is associated with significantly increased expression levels of genes (**: $p < 0.01$, Wilcoxon rank sum test). Bar plots lists the enriched GO terms for the two sets of genes, respectively. **(D)** Statistics of enhancers in Fig. 5B categorized by the chromatin state in NSCs.
